# Association Between the Circulating Level of 25-Hydroxyvitamin D and Clinical Results After Cardiac Surgery: A Meta-Analysis and Systematic Review

**DOI:** 10.3389/fcvm.2021.734504

**Published:** 2021-11-15

**Authors:** Yulin Zhang, Jiawen Li, Yu Qiu, Xue Gong, Yunru He, Peng Yue, Xiaolan Zheng, Lei Liu, Hongyu Liao, Kaiyu Zhou, Yimin Hua, Yifei Li

**Affiliations:** Key Laboratory of Birth Defects and Related Diseases of Women and Children of MOE, Department of Pediatrics, West China Second University Hospital, Sichuan University, Chengdu, China

**Keywords:** 25-(OH)-VitD, cardiac surgery, clinical outcomes, prognosis, meta-analysis

## Abstract

**Background:** Vitamin D (VitD) is an important pleiotropic hormone for organ systems. Studies have focused on the level of VitD, especially that of 25-hydroxyvitamin D (25-(OH)-VitD), in patients after cardiac surgery and the relationship between VitD deficiency and adverse outcomes, but the results have been inconsistent. We carried out a meta-analysis to evaluate differences in the 25-(OH)-VitD level before and after cardiac surgery, and evaluated the predictive value of 25-(OH)-VitD level in the clinical outcomes of patients undergoing cardiac surgery.

**Methods:** Studies related to VitD level and cardiac surgery were searched from PubMed, EMBASE, Web of Science, and Cochrane Central Register of Controlled Trials databases from inception to October 2020. We applied the Newcastle–Ottawa Scale to assess the risk of a bias in individual studies. We examined the heterogeneity and publication bias and performed subgroup analyses and sensitivity analyses.

**Results:** Fifteen studies were included in our analysis. The 25-(OH)-VitD level was significantly lower immediately after surgery [stand mean difference (SMD), 0.69; 95%CI (0.1, 1.28), *P* = 0.023] and 24-h after surgery [0.84; (0.47, 1.21), 0.000] compared with that before surgery. A higher prevalence of 25-(OH)-VitD deficiency was recorded 24 h after surgery [RR, 0.59; 95%CI (0.47, 0.73), *P* = 0.00]. Pooled results demonstrated a significant relationship between the preoperative 25-(OH)-VitD level and vasoactive-inotropic score (VIS) [SMD, −3.71; 95%CI (−6.32, −1.10); *P* = 0.005], and patients with 25-(OH)-VitD deficiency revealed a comparatively poor prognosis and severe condition after cardiac surgery [−0.80; (−1.41, −0.19), 0.01]. However, 25-(OH)-VitD deficiency was not associated with the duration of stay in the intensive care unit.

**Conclusions:** Cardiac surgery would leads to deficiency of 25-(OH)-VitD. And the preoperative and postoperative levels of 25-(OH)-VitD are associated with adverse events, which is eligible to work as an indicator to demonstrate clinical outcomes.

## Introduction

Vitamin D (VitD) has well-established roles in calcium absorption and bone mineralization (1). Exposure to sunlight and dietary intake are essential to ensure an adequate store of VitD, which functions mainly in the intestine, kidney, and bone (2). 25-hydroxyvitamin D (25-(OH)-VitD) is the active hormonal form of VitD, and is also the best way to measure the VitD level in blood. VitD deficiency is related to rickets in children and osteomalacia in adults. Supplementation with VitD can prevent bone fractures in older people effectively (3–5).

Besides its traditional role in bone mineral density, VitD might take part in cardiovascular disease by directly activating nuclear receptors in cardiomyocytes and vascular endothelial cells. Moreover, it also regulates the renin–angiotensin–aldosterone system, lipid metabolism, and energy expenditure to influence the cardiovascular system (6). VitD has been reported to suppress pro-inflammatory cytokines and promote anti-inflammatory cytokines in children and adults with congestive heart failure (7, 8). Besides, VitD may play a part in myocardial contractility, and VitD deficiency is associated with an increased risk of cardiovascular disease and adverse outcomes. VitD supplementation in patients with documented VitD deficiency is associated with improved survival (9). Recently, Turan et al. found that the VitD level was linked to several factors that might influence outcomes after cardiac surgery (10). VitD deficiency is strongly associated with an increased risk of cardiovascular events (11).

VitD deficiency is one of the most common chronic medical conditions worldwide, especially in patients with cardiovascular diseases (e.g., coronary disease, peripheral arterial disease, and heart failure). Besides, several complications might occur due to VitD loss following cardiac surgery, which contributes to poor prognosis (10, 12, 13). Cardiovascular diseases are a major cause of death in developed countries. As a consequence, >1 million cardiac-surgery procedures are carried out worldwide each year on children and adults (10). Recently, several studies have reported that after cardiac surgery in adults, such as cardiopulmonary bypass (CPB), an acute reduction in the 25-(OH)-VitD level was observed (14), and that 25-(OH)-VitD deficiency is associated with several adverse effects.

To investigate the 25-(OH)-VitD level before and after surgery and the predictive value of the 25-(OH)-VitD level in clinical outcomes after cardiac surgery, we carried out this meta-analyses to determine the changes of 25-(OH)-VitD and its prognostic value for adverse cardiovascular events after cardiac surgery. We are looking forward to help physicians to make timely and optimal clinical consideration about poor outcomes on this issue.

## Materials and Methods

This systematic review was conducted in accordance with the guideline for systematic reviews of prognostic-factor studies (15). We reported by following the PRISMA 2020 statement: an updated guideline for reporting systematic reviews (16). The study protocol is registered in the International Prospective Register of Systematic Reviews (PROSPERO).

### Search Strategy

A systematic search of PubMed, EMBASE, the Cochrane Central Register of Controlled Trials, and Web of Science databases was conducted to identify relevant studies on 10 December 2020 without date restrictions. Key search terms were (“vitamin D”) AND (“heart surgery” OR “cardiac surgery”). We searched the PubMed database using [vitamin D [MeSH Terms]) OR (vitamin D)] AND [(heart surgery [MeSH Terms]) OR (cardiac surgery) OR (heart surgery) OR (heart operation) OR (cardiosurgery)].

Two independent investigators undertook thorough literature searches, with discrepancies resolved by a third investigator in a blinded fashion. The retrieved results were de-duplicated and screened against the pre-specified eligibility criteria.

### Study Selection

Two reviewers screened the titles and abstracts of studies independently. Then, they assessed the full text of the selected studies in detail for eligibility. We excluded studies if the duplicated data for all outcomes of interest were published elsewhere, and preserved the studies that provided comparative data if there was an overlap of data between studies.

We developed inclusion criteria based on the Population, Index prognostic factor, Comparator prognostic factor, Outcome, Timing, Settings (PICOTS) framework adapted from the guideline proposed by Riley et al. (15). We defined patients as having confirmed VitD deficiency if they had a VitD concentration in serum <20 ng/mL or <50 nnmol/L irrespective of clinical signs and symptoms (17). Eligible studies had to meet the following criteria: (1) population: patients having undergone cardiac surgery and VitD test without supplementation with VitD after the surgical procedure. (2) Index prognostic factor: VitD deficiency was defined as VitD concentration in serum <50 nnmol/L irrespective of clinical signs and symptoms (17). (3) outcome: major adverse cardiovascular events (the composite of myocardial infarction, stroke, or cardiovascular death), SYNTAX score >22, maximum VIS >20, and duration of stay in the ICU. (4) time: preoperative and postoperative VitD level should be measured within a week before and after surgery. (5) Setting: in-hospital.

Inter-rater reliability for the study selection was calculated using the kappa statistic. Studies meeting any of the following criteria were excluded: (1) patients with heart disease were not related the surgery (2) Time of VitD measurement were not reported (3) The outcomes didn't meet with our definition (4) conference articles, reviews, abstracts, other non-peer-reviewed literatures or those not based on original studies.

### Data Collection and Assessment of Study Quality

Two investigators (Yulin Zhang and Jiawen Li) assessed the eligibility of reports independently at the title and abstract level. A third reviewer (Yifei Li) determined divergence according to the inclusion or exclusion criteria and quality of the reports. Studies that met all of the inclusion criteria were selected for further analyses. The baseline data from the included studies were extracted and are shown in Table 1. The quality of the included studies in the meta-analysis was assessed using the Newcastle–Ottawa Scale (NOS). In addition, studies which scored ≥5 stars were considered to have moderate-to-high methodological quality.

**Table 1:** Characteristics of involved patients with cardiac surgery in all included studies.

**References**	**Country**	**Definition of VitD deficiency**	**Study type**	**Age**	**Male (%)**	**Patients**	**Surgery method**
Rippel et al. (2)	Australia	<50 nmol/L	Prospective	8.7/9.2 m	59.2/50.6	125/85	CHD
Graham et al. (18)	America	<20 ng/ml	Prospective	8.8 d	Not report	70	CHD
McNally et al. (19)	Canada	<20 ng/ml	Prospective	8.4 m	56.9	58	CHD
Sriram et al. (20)	America	<20 ng/ml	Prospective	59/58 y	55/77	20/44	MIX
Skuladottir et al. (21)	Iceland	Not report	Prospective	64/70.5 y	78.8/81.8	52/66	MIX
Shadvar et al. (22)	Iran	Not report	Cross-sectional	8/3 y	50/50	25/25	CABG
Gode et al. (23)	Turkey	Not report	Prospective	58.4/59.1 y	67/67	75/15	CABG
Emren et al. (24)	Turkey	Not report	Prospective	61/70 y	76/72	212/71	CABG
Abou Zahr et al. (25)	America	<20 ng/ml	Prospective	71 m	50	20	CHD
Cerit et al. (26)	Cyprus	<20 ng/ml	Retrospective	63.9/67.6 y	77/35	87/41	CABG
Özsin et al. (27)	Turkey	Not report	Retrospective	58.18/61.94 y	33/37	50/50	CABG
Daie et al. (28)	Iran	<20 ng/ml	Prospective	61.19/63.89 y	68.5/62.1	127/29	CABG
Zajic et al. (29)	Austria	Not report	Prospective	67 y	19	26	MIX
Ney et al. (30)	Germany	<20 ng/ml	Prospective	67.27/70.06 y	72.2/72.7	74/18	MIX
Cerit and Cerit (1)	Cyprus	<20 ng/ml	Prospective	61.3/74.9 y	44.7/43.9	47/98	CABG
Dohain et al. (31)	Egypt	<20 ng/ml	Prospective	14.4 months	41	69	CHD

### Outcome Measures

First, we collected the VitD level of patients before, immediately after, and 24-h after undergoing cardiac surgery from correlational studies. Then, we compared the outcomes between the VitD-deficient group and VitD-sufficient group.

- The primary endpoints includes the occurrence of major adverse cardiovascular events, the composite of myocardial infarction, stroke, or cardiovascular death.
- The secondary endpoints includes arrhythmia, SYNTAX score and maximum VIS after surgery, and ICU stay duration.

### Publication Bias

A publication bias was tested using Egger's regression and funnel graph by STATA 15.1 (State, College Station, TX, USA). Each dot represents a study in the meta-analysis and asymmetry of distribution of dots indicates a potential publication bias. A quantified result of *P* < 0.05 in Egger's test indicated that a publication bias might be present.

### Heterogeneity

Heterogeneity in the pooling sensitivity and specificity was examined using the Q-test and was deemed to be significant if *P* < 0.10 in these qualitative tests. The *I*^2^ test was also carried out in each pooling analysis to estimate quantitatively the proportion of total variation across studies that were due to heterogeneity rather than chance. *I*^2^ can range from 0 to 100%, and *I*^2^ >50% suggests significant heterogeneity.

### Sensitivity Analyses

Sensitivity analyses were conducted (using STATA 15.1) for each study to determine if a single study incurred undue weight in the meta-analysis fixed/random-effects estimates.

### Statistical Analyses

Analyses were undertaken for adjusted and unadjusted estimates. Adjusted estimates were utilized primarily for reporting and interpretation of results (38). The pooled effects of dichotomous outcomes were converted to risk ratios (RRs) along with their 95% confidence intervals (CIs). Quantitative synthesis was first conducted by comparing VitD-deficient patients with VitD-sufficient patients or the highest vs. lowest categories of VitD using the generic inverse variance method with the DerSimonian–Laird random-effects model (15). Continuous outcome variables are expressed as the mean ± SD. All pooled effects are presented as forest maps. Quality of evidence was assessed by the modified Grading of Recommendations Assessment, Development, and Evaluation system (GRADE) by consensus among the authors (39, 40). If pooled effect sizes with great heterogeneity comprised >5 studies, subgroup analyses were carried out based on the study design, study location, sample size, risk of a bias, type of effect size, and surgical method. Conversely, sensitivity analyses were conducted by leave-one-out analysis and the exclusion of studies with a high risk of a bias. GRADE was used to evaluate the overall quality of the evidence for each outcome, which ranged from high quality to very low quality, and was based on five domains: limitations of design; inconsistency of results; indirectness; imprecision; other factors (e.g., a publication bias) (Table 2).

**Table 2:** Quality assessment of studies using criteria adapted from Newcastle-Ottawa quality assessment scale (32, 33).

	Representative^*^	**Reliably measured VitD^†^**	**Comparable on confounders^‡^**	**Adequate outcome and follow up^§^**	**Overall quality rating^¶^**
Dohain et al. (31)	0	1	1	Not reported	Low
Abou Zahr et al. (25)	0	1	0	1	Low
McNally et al. (19)	0	1	0	0	Low
Rippel et al. (2)	1	1	1	1	High
Graham et al. (18)	1	1	1	1	High
Zarei et al. (34)	1	1	2	1	High
Zajic et al. (29)	0	1	0	1	Low
Ney et al. (30)	1	1	1	1	Low
Özsin et al. (27)	1	1	1	1	High
Obeid et al. (35)	1	0	0	1	Low
Daie et al. (28)	0	0	0	Not reported	Low
Cerit et al. (36)	1	1	0	Not reported	Low
Cerit et al. (26)	1	1	0	0	Low
Zittermann et al. (37)	1	1	1	1	High
Skuladottir et al. (21)	0	1	2	1	Average
Shadvar et al. (22)	0	0	0	1	Low
Gode et al. (23)	0	1	2	1	High
Sriram et al. (20)	0	0	1	1	Low
Emren et al. (24)	1	1	1	1	High
Zittermann et al. (5)	1	1	2	1	High

* *The representativeness criterion was met when ≥80% of patients after cardiac surgery eligible were invited and 80% agreed to participate, or when sample size >300 (1 point)*.

† *The reliability criterion was met when reliable and valid methods were used to assess VitD level (1 point)*.

‡ *The comparability criterion was met when studies showed evidence that at study entry the patients with or without events were equivalent on the prognostic indicators of age, previous use of d-dimer supplement, surgical method, and comorbidity (2 points) or comparable on at least two of these indicators (1 point)*.

§ *The quality of outcome and follow-up criterion was met when the completion rate (agreed to participate/analyzed) for patients undergoing the cycle was ≥80% (1 point)*.

¶ *The overall quality rating was low (0–2 points), average (3 points), or high (4 or 5 points)*.

## Results

### Search Results

**Figure 1:**
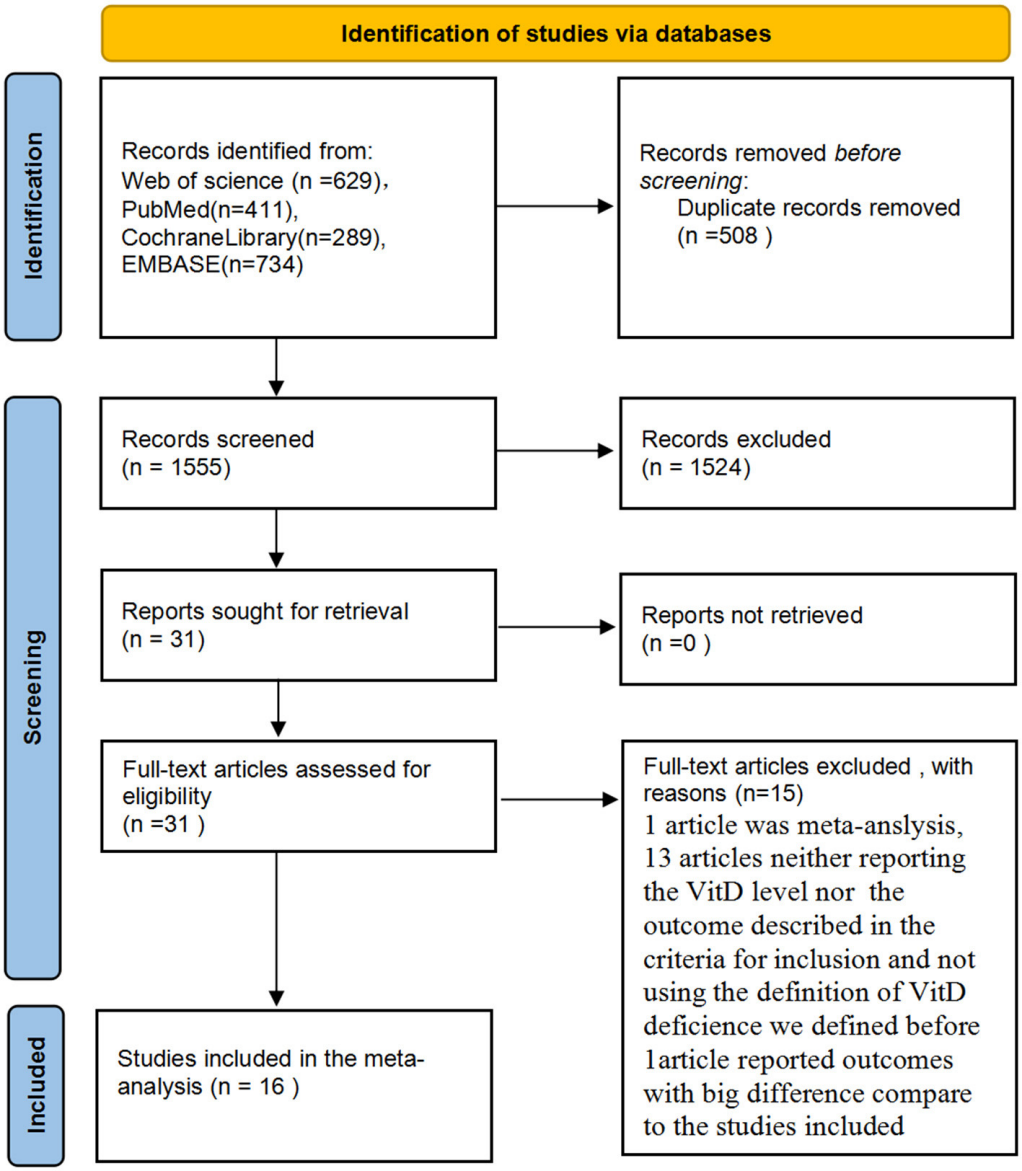
Preferred reporting items for systematic reviews and meta-analyses (PRISMA) flow diagram for study identification and selection.

Initially, 2,063 potentially relevant articles were retrieved by our search method. Of these, 31 articles were considered to be of interest after reading the title and abstract. However, 15 articles were excluded by reading the complete articles due to: article type (*n* = 1); absence of measurement of VitD levels at required time points or the outcomes described in the criteria of inclusion (*n* = 13); not using the standard definition of VitD deficiency (*n* = 1). Ultimately, 16 reports (1, 2, 18–31) were included in the meta-analysis (Figure 1). No reports from China met the inclusion criteria.

### Study Characteristics

The characteristics of the involved patients in all included studies are presented in Table 1. The sixteen published reports (1, 2, 18–31) enrolled a total of 1,785 patients (children and adults) from 10 countries. Five studies (2, 18, 19, 25, 31) focused on children with congenital heart disease and 11 studies (1, 20–24, 26–30) were on adults, of which seven studies (1, 22–24, 26–28) reported patients undergoing coronary artery bypass surgery (CABG). Eleven studies (1, 2, 18, 19, 21, 23–26, 29, 31) reported a detailed method used to measure the serum concentration of VitD and 10 studies (1, 2, 18–20, 25, 26, 28, 30, 31) reported a clear definition of VitD deficiency as 25-(OH) VitD <20 ng/ml or <50 nmol/L. About half of the included studies yielded a high risk of a bias and the other half were at low risk according to the NOS (Table 2).

### Outcomes

Table 3 presents the pooled effect sizes and 95%CIs for the synthesis analysis, and GRADE level of certainty.

**Table 3:** The summary of GRADE assessment.

**No of studies**	**Certainty assessment**						**Effect**			**Certainty**	**Importance**
	**Study design**	**Risk of bias**	**Inconsistency**	**Indirectness**	**Imprecision**	**Other considerations**	**No of events**	**No of individuals**	**Rate (95% CI)**		
**Comparison of 24 h post-operational and pre-operational VitD level of cardiac surgery**											
8	Observational studies	Serious^a^	Serious^b^	Serious^c^	Not serious	None	-	453	SMD 0.84 SD (0.47–1.21)	⊕◯◯◯ Very low	Critical
**Comparison of immediately post-operational and pre-operational VitD level of cardiac surgery**											
6	Observational studies	Serious^a^	Serious^b^	Serious^c^	Not serious	None	-	335	SMD 0.69 SD (0.1–1.28)	⊕◯◯◯ Very low	Important
**Comparison of the rates of pre-operational and 24 h post-operational VitD deficiency**											
3	Observational studies	Serious^a^	Not serious	Serious^c^	Not serious	None	344	441	Event rate 191.3 per 1,000 (110.5 to 252.8)	⊕⊕◯◯ Low	Not Important
**Comparison of post-operational VitD level with and without severe outcomes**											
8	Observational studies	Serious^a^	Serious^b^	Serious^c^	Not serious	None	-	1,070	SMD −0.87 SD (−1.41 to −0.09)	⊕◯◯◯ Very low	Important
**Comparison of maximum VIS between groups of low and high post-operational VitD levels**											
3	Observational studies	Serious^a^	Serious^b^	Not serious	Serious	None	-	337	SMD −3.71 SD (−6.32 to −1.1)	⊕◯◯◯ Very low	Critical
**Pooled effect sizes of rate of pre-operational and 24h post-operational VitD deficiency**											
3	Observational studies	Serious ^a^	Not serious	Serious^c^	Not serious	None	244	441	event rate 181 per 1000 (117.4 to 233.1)	⊕⊕◯◯ Low	Important
**Comparison of post-operational VitD levels between patients with or without ICU admission**											
4	Observational studies	Serious^a^	Serious^b^	Serious^c^	Not serious	None	-	401	SMD −0.53 SD (−1.16 to −0.09)	⊕◯◯◯ Very low	Important

a *Most of included studies were low-quality in this outcome according to NOS*.

b *The I-square value of this outcome was more than 80% and p value of chi-square Q test was <0.05. The effect sizes of included studies were in the same direction but widely varied*.

c *The source of participants, measurement methods of VitD levels and surgical methods were different among included studies*.

### Assessment of the 25-(OH)-VitD Level

Six studies (18, 19, 25, 29–31) provided data on the VitD level preoperatively, immediately after surgery, and 24 h after surgery, and one study (21) provided data only on the VitD level preoperatively and 24 h after surgery. Furthermore, four studies (18, 19, 25, 31) reported the VitD level in children with congenital heart disease and three studies (21, 29, 30) were in adults. However, two studies (29, 30) did not mention the way to measure the serum 25-(OH)-VitD concentration. Three studies in adults (21, 29, 30) reported on patients who underwent types of cardiac surgery other than CABG.

**Figure 2:**
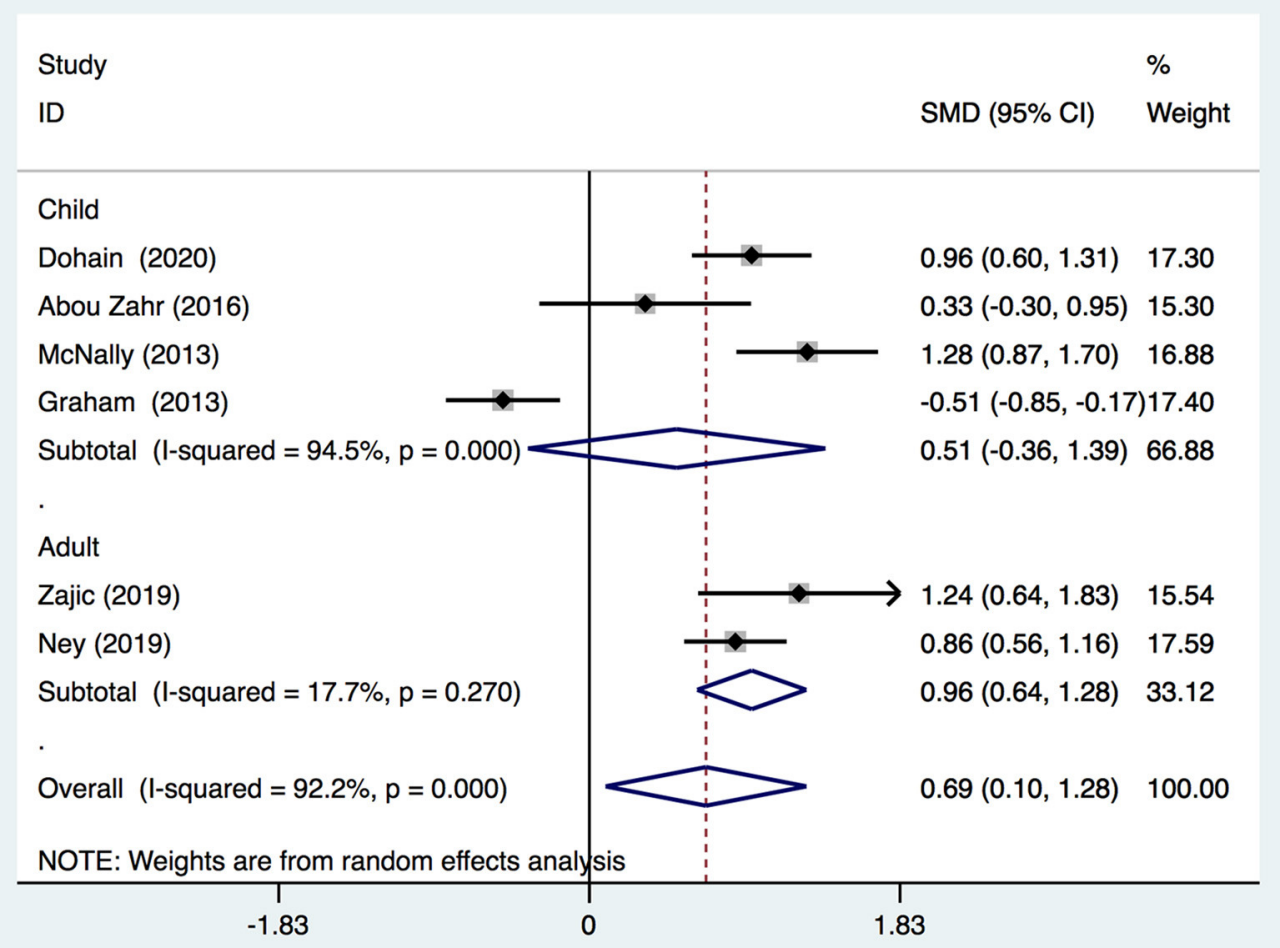
Forest plot for comparison of the VitD level pre-operation and immediately post-operation identified in the meta-analysis of six trials using random-effect model. Only the first author of each study is given. Test for overall effect, *z* = 2.28, *P* = 0.02; test for heterogeneity, *I*^2^ = 92.2%, *P* = 0.000. SMD, standard mean difference. CI, confidence interval.

**Figure 3:**
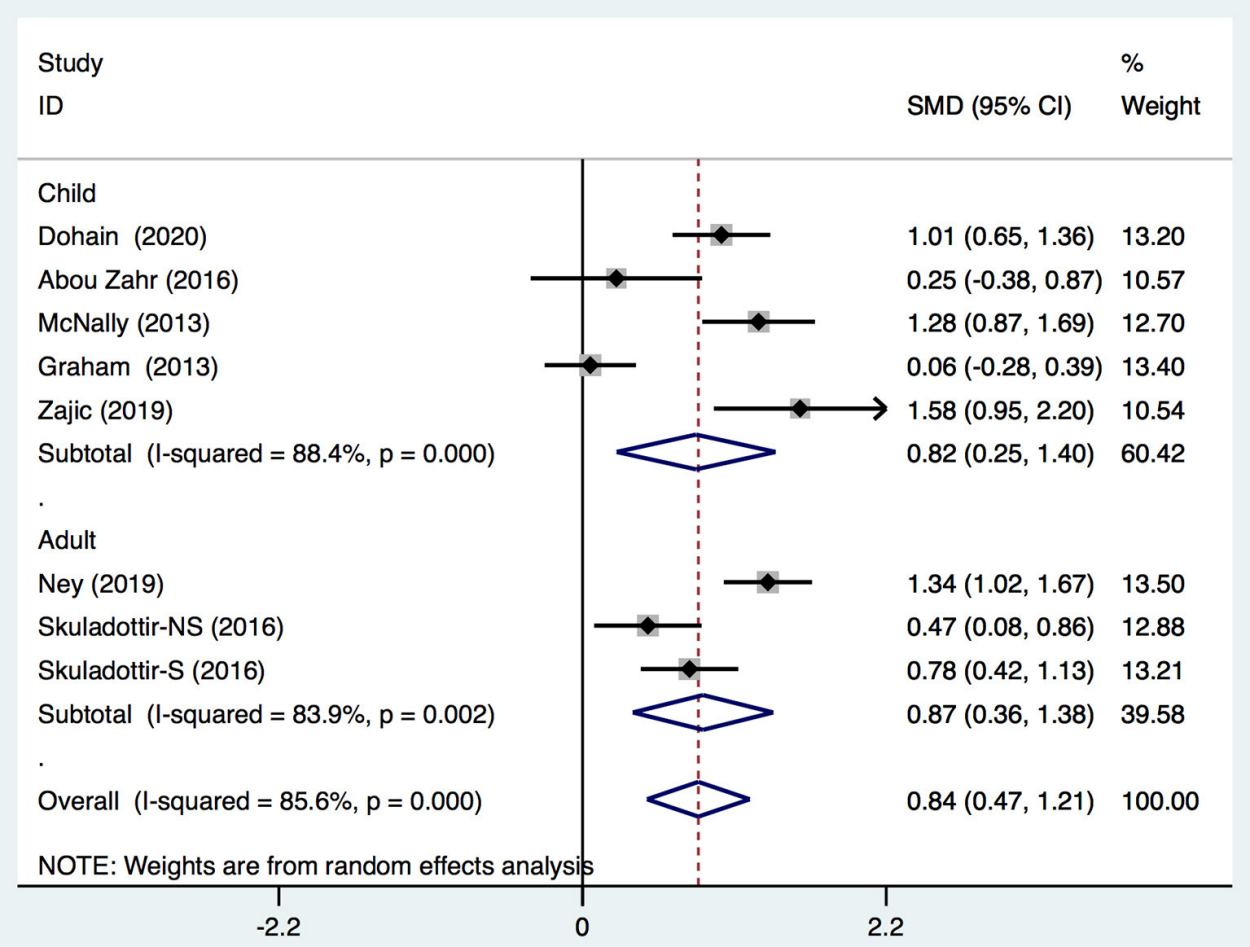
Forest plot for comparison of the VitD level preoperation and 24 h postoperation identified in the meta-analysis of eight trials using random-effect model. Only the first author of each study is given. Test for overall effect, *z* = 4.42, *P* = 0.000; test for heterogeneity, *I*^2^ =85.6%, *P* = 0.000. SMD, standard mean difference. CI, confidence interval.

We demonstrated that the VitD level decreased significantly immediately after cardiac surgery [stand mean difference (SMD), 0.69; 95%CI (0.1, 1.28); *P* = 0.000] (Figure 2) or 24 h after cardiac surgery [SMD, 0.84; 95%CI (0.47, 1.21); *P* = 0.000] (Figure 3), though all models yielded substantial heterogeneity (*I*^2^ = 92.2% for immediately after surgery; *I*^2^ = 85.6% for 24 h after surgery). Subgroup analyses based on enrolled patients (adults or children) were partially consistent with the overall effects. Immediately after surgery, adult patients demonstrated a significant reduction in the 25-(OH)-VitD level [SMD 0.96, 95%CI (0.64, 1.28), *I*^2^ = 17.7%], whereas child patients had a normal level [SMD 0.51, 95%CI (−0.36, 1.39), *I*^2^ = 94.5%]. Among data from adults, there was no heterogeneity, and the pooled heterogeneity was considered to originate from child-based studies. At 24-h after cardiac surgery, results from children and adults remained consistent with an overall significant reduction. However, heterogeneities were also demonstrated upon analyses of both subgroups. Egger's test presented an absence of a publication bias with *P* = 0.713 and *P* = 0.849 for measurements immediately after and 24-h after cardiac surgery, respectively (Supplementary Figures 1A,B).

**Figure 4:**
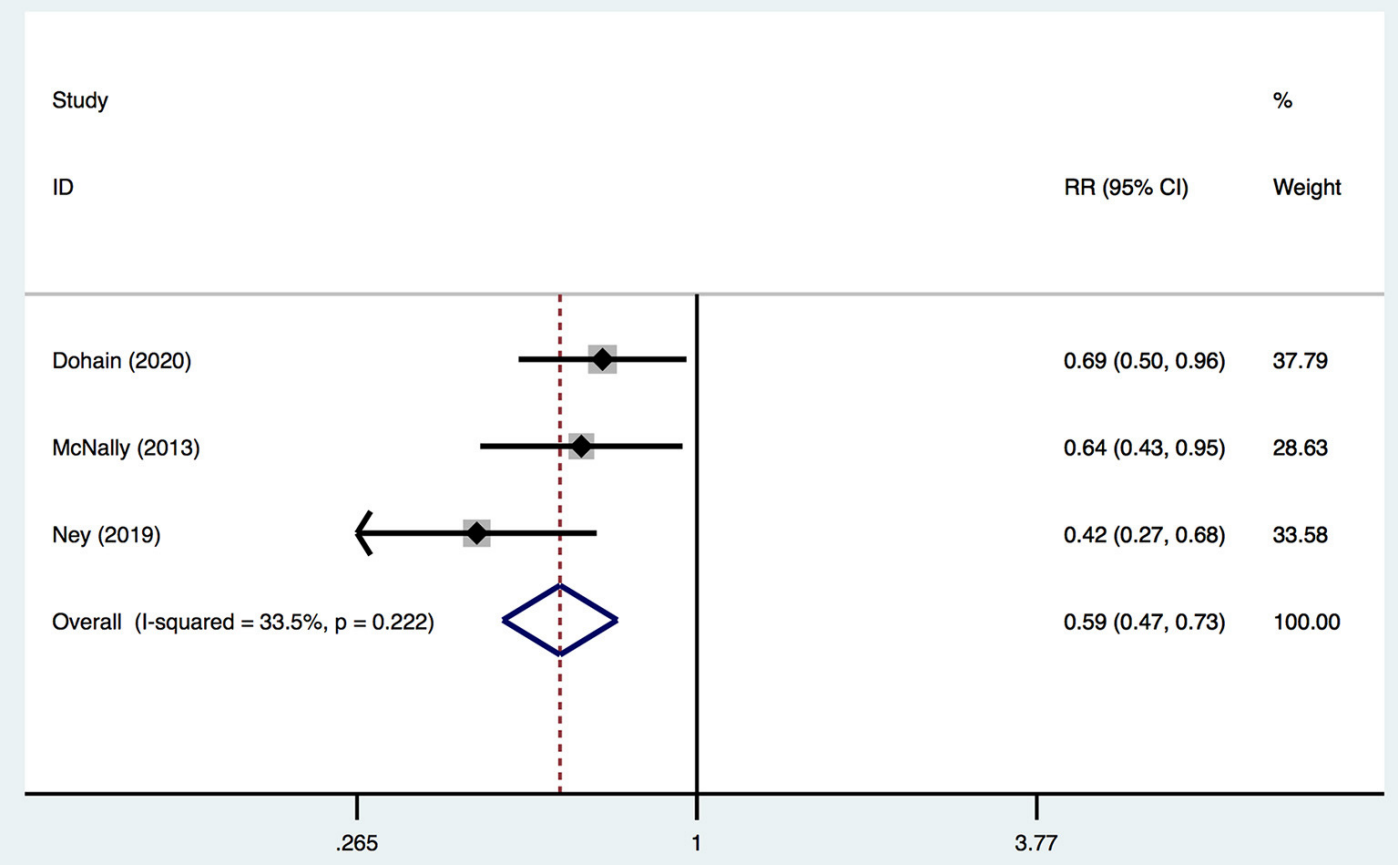
Forest plot for RRs of VitD deficiency rate of pre- and 24-h post-cardiac surgery identified in the meta-analysis of three trials using fixed-effect model. Only the first author of each study is given. Test for overall effect, *z* = 4.69, *P* = 0.00; test for heterogeneity, *I*^2^ = 33.5%, *P* = 0.22. RR, risk ratio. CI, confidence interval.

Moreover, three studies reported adjusted RRs of VitD deficiency before and 24 h after cardiac surgery. Pooled data demonstrated that cardiac surgery was negatively associated with the VitD level (RR 0.59; 95%CI (0.47, 0.73) (Figure 4) without significant heterogeneity (*I*^2^ = 33.5%). Egger's test presented an absence of a publication bias with *P* = 0.235 (Supplementary Figure 1C).

### Severe Events

**Figure 5:**
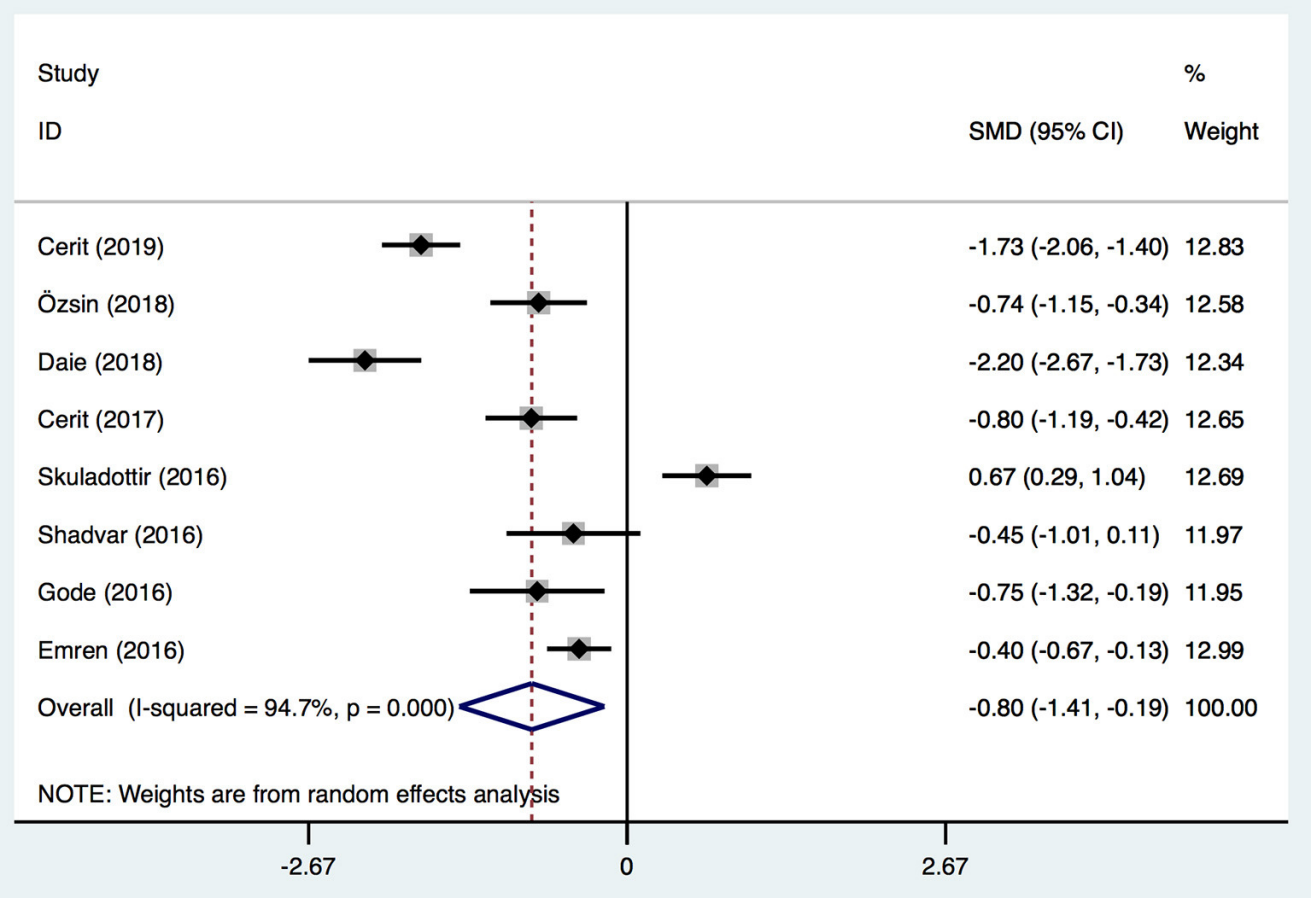
Forest plot for the relationship between VitD level and severe outcomes post-operation identified in the meta-analysis of eight trials using random-effect model. Only the first author of each study is given. Test for overall effect, *z* = 2.58, *P* = 0.01; test for heterogeneity, *I*^2^ = 94.7%, *P* = 0.00. SMD, standard mean difference. CI, confidence interval.

Eight studies (1, 21–24, 26–28) in our meta-analysis reported postoperative severe events and VitD level. Severe events included postoperative atrial fibrillation (Po-AF) and high SYNTAX score. The latter is an angiographic grading tool to evaluate the complexity and extensity of coronary artery disease (CAD). All lesions causing ≥50% of stenosis in a coronary artery of diameter ≥1.5 mm were included in calculation of the SYNTAX score, and the latter was divided into two groups: high (≥23) and low (<23) (1). AF was confirmed by 12-lead electrocardiography. Three studies (23, 27, 28) described AF as irregular, fast oscillations or fibrillary waves instead of regular P waves at electrocardiography and a range of ventricular rates between 90 and 170 bpm. An AF episode longer than 5 min or which necessitated therapy for hemodynamic instability was accepted as Po-AF. However, two studies (21, 26) did not report the criterion of AF, and one study (24) did not report the standard of Po-AF clearly. Another study (22) defined neither AF nor Po-AF precisely. All these definitions were considered to represent AF or Po-AF in the analyses. In total, the VitD level was decreased significantly after surgery in the severe group [SMD, −0.8; 95%CI (−1.41, −0.19); *P* = 0.01] (Figure 5) with significant heterogeneity (*I*^2^ = 94.7%). Egger's test revealed an absence of a publication bias with *P* = 0.737 (Supplementary Figure 1D).

### Maximum VIS

**Figure 6:**
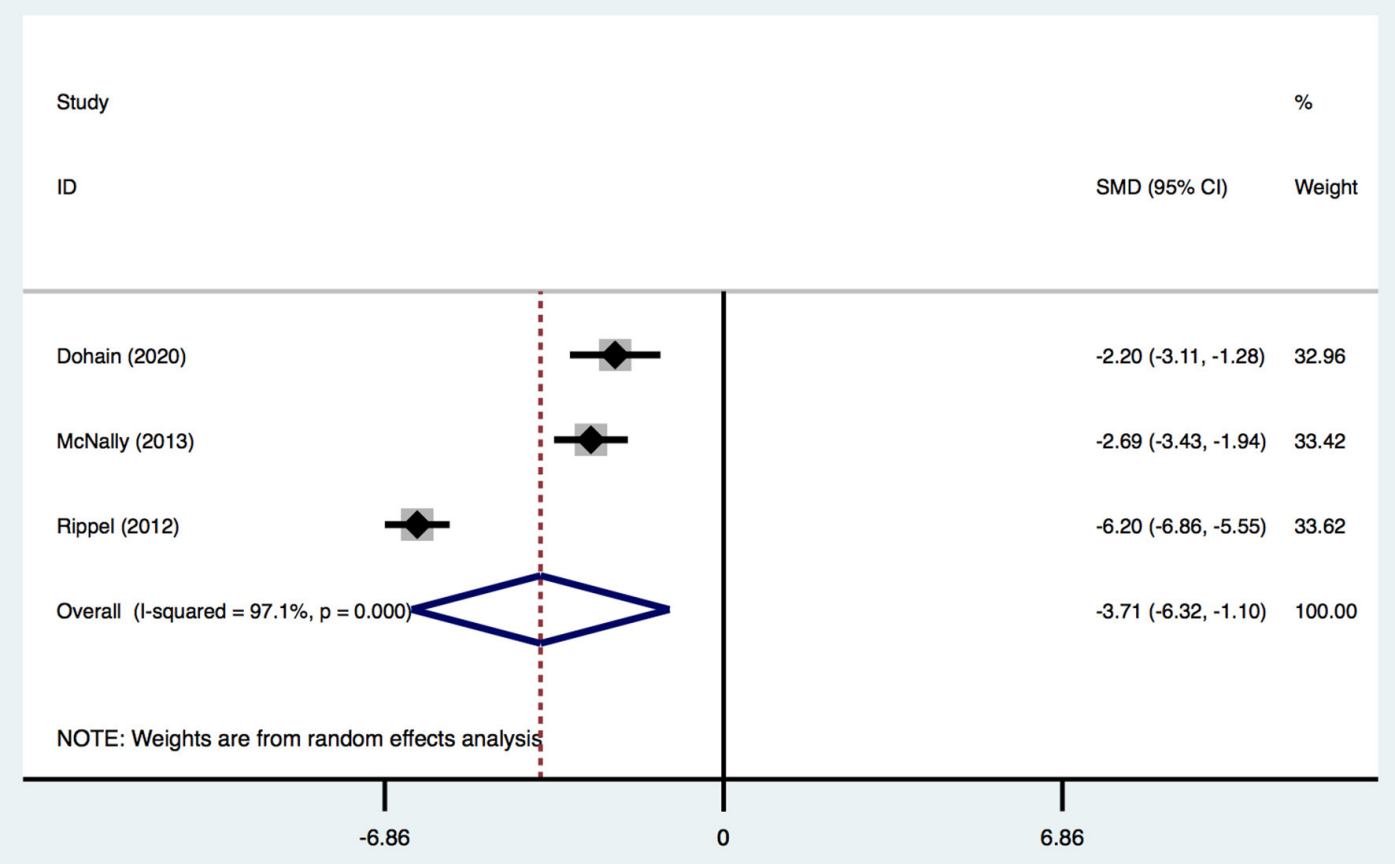
Forest plot for the relationship between VitD level and maximum VIS in the meta-analysis of three trials using random-effect model. Only the first author of each study is given. Test for overall effect, *z* = 2.78, *P* = 0.005; test for heterogeneity, *I*^2^ = 97.1%, *P* = 0.000. SMD, standard mean difference. CI, confidence interval.

Three studies (2, 19, 31) provided data on the maximum VIS and VitD level. The VIS was calculated by various equations in different studies. One study (31) calculated it using the following equation with the drug dose in μg/kg/min: (dopamine + dobutamine) + (milrinone × 10) + (epinephrine × 100) + (norepinephrine × 100). One study (19) used the following equation with the drug dose in ICU: dopamine (^*^1) + dobutamine (×1) + amrinone (×1) + milrinone (×15) + epinephrine (×100) + norepinephrine (×100). Another study (2) did not report the specific calculation of VIS, and none of the studies reported the definition of high VIS and low VIS. Given this information and the limited number of related studies, we regarded these definitions as being equivalent. Overall, children undergoing cardiac surgery with a lower postoperative VitD level had a significantly higher VIS [SMD, −3.71; 95%CI (−6.32, −1.10); *P* = 0.005, *I*^2^ = 97.1%] (Figure 6) than that of patients with normal VitD level. Egger's test revealed an absence of a publication bias with *P* = 0.368 (Supplementary Figure 1E).

### Duration of ICU Stay

**Figure 7:**
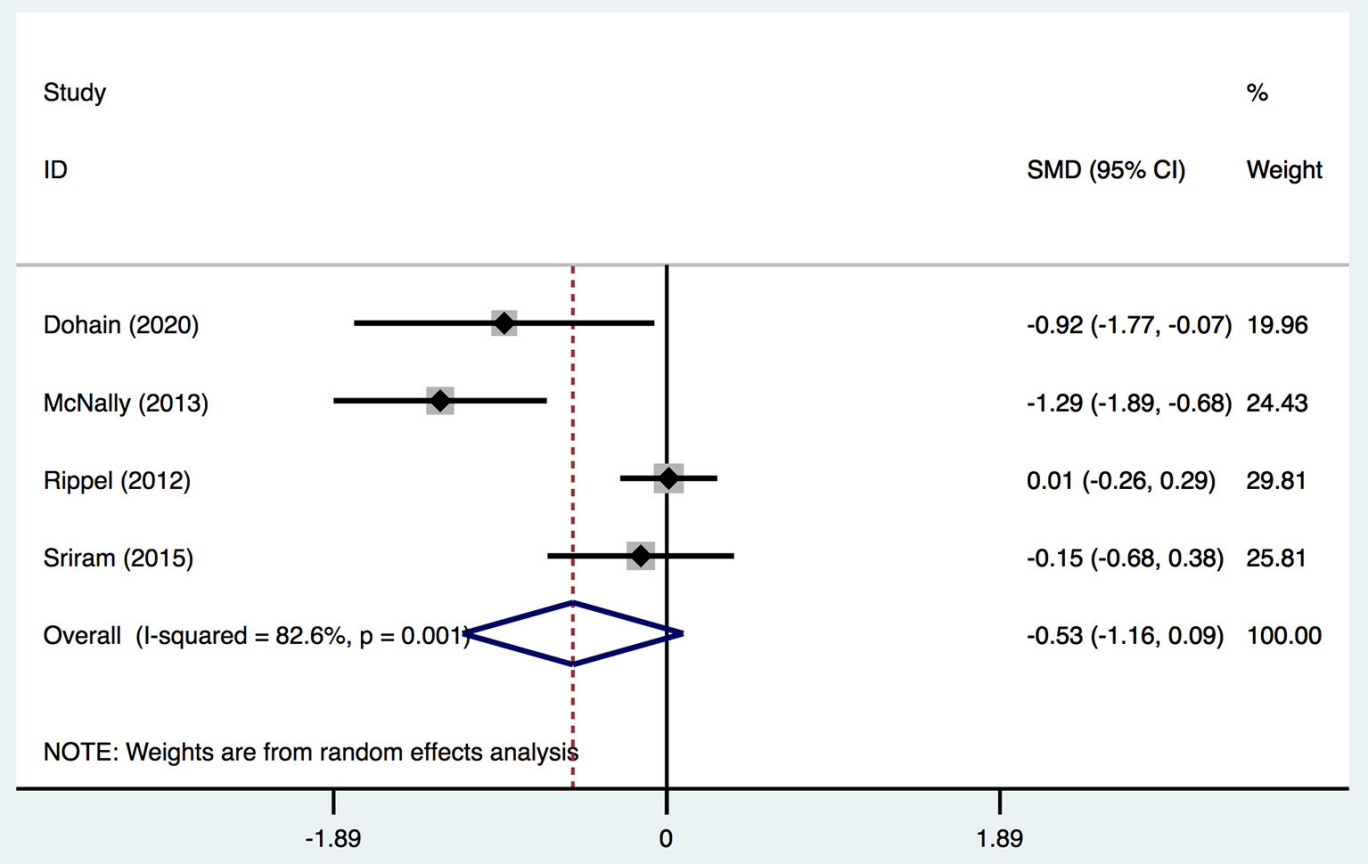
Forest plot for the relationship between VitD level and ICU stay duration in the meta-analysis of four trials using random-effect model. Only the first author of each study is given. Test for overall effect, *z* = 1.67, *P* = 0.096; test for heterogeneity, *I*^2^ =82.6%, *P* = 0.001. SMD, standard mean difference. CI, confidence interval.

Four studies (2, 19, 20, 31) provided data on the duration of ICU stay, and classified patients into two groups: VitD- sufficient and VitD-deficient. All studies defined VitD deficiency using this standard. In total, there was no significant difference in the duration of ICU stay [SMD, −0.53; 95%CI (−1.6, 0.09); *P* = 0.096] between the two groups (Figure 7). Egger's test revealed an absence of a publication bias with *P* = 0.160 (Supplementary Figure 1F).

### Sensitivity Analysis

**Figure 8:**
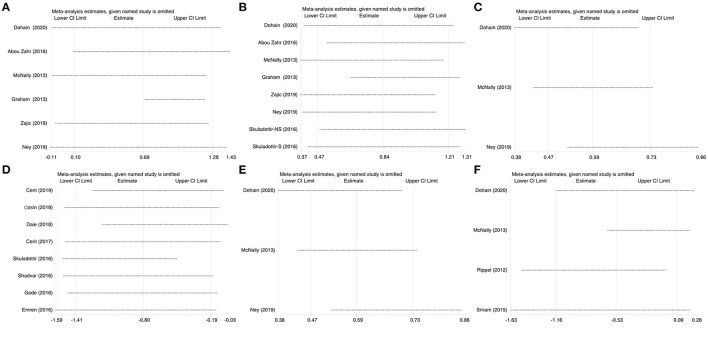
Sensitivity analysis of the individual trials on the results. **(A)** For the VitD level preoperative and immediate post cardiac surgery; **(B)** For the VitD level preoperative and 24-h post cardiac surgery; **(C)** For the RRs of VitD deficiency rate of pre- and 24-h post-cardiac surgery; **(D)** For the relationship between VitD level and severe events; **(E)** For the relationship between VitD level and maximum VIS; **(F)** For the relationship between VitD level and ICU stay duration.

We systematically and qualitatively analyzed the sensitivity across the included studies to determine the influence of individual studies on the results (Figure 8). We did not detect a significant impact from a single study. We confirmed the direction of the results except for one study (Ripple et al.) in analyses of the relationship between the duration of ICU stay and serum level of VitD (Figure 7). After excluding that study, the result was opposite and identified a significant difference in the duration of ICU stay [SMD, −0.76; 95%CI (−1.52, −0.012); *P* = 0.046] between groups.

## Discussion

There are concerns for VitD deficiency in infants, children, and adolescents because of the limited natural dietary sources of VitD and insufficient sunshine exposure for the cutaneous synthesis of VitD in some areas. Emerging evidence supports a potential role for VitD in maintaining innate immunity and preventing diseases such as diabetes mellitus, cancer, severe asthma, acute respiratory infection, and cardiomyopathy (41–45). An increasing number of studies have reported that VitD deficiency is prevalent in patients, especially those who have undergone cardiac surgery. The prevalence of VitD deficiency in children with congenital heart diseases has been reported to range from 40 to 84% (18, 19, 25). However, among critically ill children, the prevalence of VitD deficiency is not reduced further, and has been reported to range from 35 to 70% (2, 19, 46). We found that cardiac surgery reduced the VitD level, which was negatively associated with adverse outcomes, such as postoperative severe events and a higher SYNTAX score and VIS. These findings are consistent with those in other studies, and may be explained (at least in part) by the high prevalence of postoperative VitD deficiency conferred by borderline-normal or low preoperative levels and an acute intraoperative decline. McNally et al. (19) described that this decline occurs immediately following CPB initiation, which suggests a dilution effect from the prime volume. Another reason for this acute intraoperative decline could be 25-(OH)-VitD absorption on the CPB tubing or oxygenator membrane.

Among the studies (including our meta-analysis), only Ney et al. (30) could not find a reduction in the VitD level after cardiac surgery. Other studies demonstrated that the postoperative VitD level was decreased significantly compared with that before surgery. VitD deficiency could affect cardiovascular health negatively through calcium homeostasis and influence cardiomyocyte and endothelial function through cellular VitD receptors, thereby resulting in postoperative adverse outcomes (47, 48) and various cardiovascular diseases (49–52). Several studies have found that a lower serum VitD level is an independent predictor of CAD (53, 54). Recently, several pathophysiological mechanisms have been proposed to explain the association between VitD deficiency and AF. One of the most important mechanisms is activation of the renin–angiotensin–aldosterone system. A meta-analysis conducted by Öztürk et al. (55) found a significant relationship between the preoperative level of VitD and AF after cardiac surgery (*p* < 0.05). Those results were consistent with those of our study, but pooled results had great heterogeneity. Other studies have reported postoperative adverse outcomes, such as intubation duration (2, 5, 19, 23, 31, 37), hospitalization duration (5, 20, 31, 37), mortality (2, 31, 34, 35), and found a significant association with the VitD level. However, pooled analysis was prohibited by few studies. According to our results, the level of VitD do identify the higher possibilities of adverse cardiovascular events and VIS, which would be involved different treatment strategies. Generally, such events lead to longer duration of ICU stay. However, the more positive therapeutic strategies would reduce the difference on ICU stay from adverse prognosis. Although, our analysis demonstrated a negative results, but remained a critical P value as 0.096. Moreover, sensitivity analysis showed one study exclusion would reach a significance results which sufficient VitD help to reduce the duration of ICU stay. So that, we believe the level of VitD would affect the ICU stay but require more studies to be included.

We wished to investigate if preoperative VitD supplementation affects outcomes. Our database search revealed a recent randomized placebo-controlled trial of VitD supplementation in 80 infants with congestive heart failure (8): it showed improvements in clinical symptoms and cardiac functions. Cerit et al. (36) demonstrated an obvious improvement in PO-AF prevalence in patients with VitD deficiency after cardiac surgery between a VitD-supplementation group and control group (18 vs. 29%) (*P* = 0.02). In addition, VitD treatment may improve the inflammatory status, reduce the apoptosis rate, and regulate the renin–angiotensin–aldosterone system and electromechanical system of the left atrium (36, 56).

Our meta-analysis had four main limitations. First, only articles written in English were included; therefore, selective, and reporting biases might have been induced. Second, meta-analyses could not be interpreted for many outcomes because of the significant clinical heterogeneity across included studies. Third, definitions of outcomes (e.g., AF, Po-AF, high VIS) in each study were applied in our meta-analysis, and inconsistencies in these definitions might have introduced biases in the pooled results. Fourth, although most of the definitions of VitD deficiency in the included studies were identical to those in the inclusion criteria, the measurement methods varied across studies. Such variation might have induced biases in our results. The scarcity of included studies and undetailed description of confounding factors hindered exploration of the source of heterogeneity by subgroup analysis and meta-regression. Therefore, caution should be exercised in interpreting our results or in generalizing these results in clinical practice. Further studies with a higher level of evidence (especially randomized controlled trials) are needed to investigate the association between the VitD level and prognosis after cardiac surgery.

## Conclusions

A reduction in the VitD level is prevalent in patients who have undergone cardiac surgery. The VitD level is an important predictor for a poor prognosis. VitD could be an indicator to underline the risks of cardiac surgeries. Besides, more prospective studies are needed to explore the prognostic value of the VitD level in patients with cardiovascular disease undergoing cardiac surgery. And trails on peri-operational VitD supplementation are still necessary to identify the advantages of VitD administration.

## Data Availability Statement

The original contributions presented in the study are included in the article/Supplementary Material, further inquiries can be directed to the corresponding authors.

## Author Contributions

YL conceived of the presented idea. JL, YZ, and YL summarized the reference and draft the manuscript. YZ and JL draft the table. YQ, XG, YuH, PY, XZ, LL, and HL participate in interpreting the results from this analysis. KZ and YiH supervised the project. YL approved the final version of the manuscript. All authors contributed to the article and approved the submitted version.

## Funding

All phase of this study was supported by a National Key R&D Program of China (2018YFC1002301). The funders had no role in study design, data collection and analysis, decision to publish, or preparation of the manuscript.

## Conflict of Interest

The authors declare that the research was conducted in the absence of any commercial or financial relationships that could be construed as a potential conflict of interest.

## Publisher's Note

All claims expressed in this article are solely those of the authors and do not necessarily represent those of their affiliated organizations, or those of the publisher, the editors and the reviewers. Any product that may be evaluated in this article, or claim that may be made by its manufacturer, is not guaranteed or endorsed by the publisher.

## Supplementary Material

Supplementary Figure 1 Egger's publication bias plots for the assessment of potential publication bias. Each dot represents each study in the meta-analysis. **(A)** For the VitD level preoperative and immediate post cardiac surgery, and the Egger's plot did not show significant asymmetry (*P* = 0.713, *t* = 0.39, 95%CI (−15.13, 20.14), **(B)** for the VitD level preoperative and 24-h post cardiac surgery, the Egger's plot did not show marked asymmetry, *P* = 0.849, *t* = 0.2, 95%CI (−10.37, 12.21), **(C)** for the RRs of VitD deficiency rate of pre- and 24-h post-cardiac surgery, the Egger's plot did not show significant asymmetry, *P* =0.235, *t* = −2.58, 95%CI (−38.02, 25.19), **(D)** for the relationship between VitD level and severe events, the Egger's plot did not show significant asymmetry, *P* = 0.737, *t* = −0.35, 95%CI (−19.55, 14.64), **(E)** for the relationship between VitD level and maximum VIS, the Egger's plot did not show significant asymmetry, *P* = 0.368, *t* = 1.54, 95%CI (−221.27, 282.09), **(F)** for the relationship between VitD level and ICU stay duration. This Egger's plot indicates no publication bias with a *P* > 0.05. RR, risk ratio. CI, confidence interval.

## References

[1] Cerit L Cerit Z. Vitamin D deficiency is not associated with higher levels of SYNTAX score. Braz J Cardiovasc Surg. (2019) 34:57–61. 10.21470/1678-9741-2018-0178 30810675 PMC6385829

[2] Rippel C South M Butt WW Shekerdemian LS. Vitamin D status in critically ill children. Intensive Care Med. (2012) 38:2055–62. 10.1007/s00134-012-2718-6 23052958

[3] Bischoff-Ferrari HA Dawson-Hughes B Staehelin HB Orav JE Stuck AE Theiler R . Fall prevention with supplemental and active forms of vitamin D: a meta-analysis of randomised controlled trials. BMJ. (2009) 339:b3692. 10.1136/bmj.b3692 19797342 PMC2755728

[4] Bischoff-Ferrari HA Willett WC Orav EJ Lips P Meunier PJ Lyons RA . A pooled analysis of vitamin D dose requirements for fracture prevention. N Engl J Med. (2012) 367:40–9. 10.1056/NEJMoa1109617 22762317

[5] Zittermann A Kuhn J Dreier J Knabbe C Gummert JF Börgermann J. Vitamin D status and the risk of major adverse cardiac and cerebrovascular events in cardiac surgery. Eur Heart J. (2013) 34:1358–64. 10.1093/eurheartj/ehs468 23315905

[6] Al Mheid I Quyyumi AA. Vitamin D and cardiovascular disease: controversy unresolved. J Am College Cardiol. (2017) 70:89–100. 10.1016/j.jacc.2017.05.031 28662812

[7] Schleithoff SS Zittermann A Tenderich G Berthold HK Stehle P Koerfer R. Vitamin D supplementation improves cytokine profiles in patients with congestive heart failure: a double-blind, randomized, placebo-controlled trial. Am J Clin Nutr. (2006) 83:754–9. 10.1093/ajcn/83.4.754 16600924

[8] Shedeed SA. Vitamin D supplementation in infants with chronic congestive heart failure. Pediatr Cardiol. (2012) 33:713–9. 10.1007/s00246-012-0199-6 22349668

[9] Vacek JL Vanga SR Good M Lai SM Lakkireddy D Howard PA. Vitamin D deficiency and supplementation and relation to cardiovascular health. Am J Cardiol. (2012) 109:359–63. 10.1016/j.amjcard.2011.09.020 22071212

[10] Turan A Grady M You J Mascha EJ Keeyapaj W Komatsu R . Low vitamin D concentration is not associated with increased mortality and morbidity after cardiac surgery. PLoS ONE. (2013) 8:e63831. 10.1371/journal.pone.0063831 23724006 PMC3665712

[11] Peterlik M Cross HS. Vitamin D and calcium deficits predispose for multiple chronic diseases. Eur J Clin Invest. (2005) 35:290–304. 10.1111/j.1365-2362.2005.01487.x 15860041

[12] Braun LA Spitzer O Levkovich B Bailey M Stanguts C Hose L . Prevalence of vitamin D deficiency prior to cardiothoracic surgery. Heart Lung Circ. (2014) 23:978–80. 10.1016/j.hlc.2014.03.014 24996389

[13] McNally JD O'Hearn K Lawson ML Maharajh G Geier P Weiler H . Prevention of vitamin D deficiency in children following cardiac surgery: study protocol for a randomized controlled trial. Trials. (2015) 16:402. 10.1186/s13063-015-0922-8 26353829 PMC4564959

[14] Krishnan A Ochola J Mundy J Jones M Kruger P Duncan E . Acute fluid shifts influence the assessment of serum vitamin D status in critically ill patients. Crit Care. (2010) 14:R216. 10.1186/cc9341 21110839 PMC3219984

[15] Riley RD Moons KGM Snell KIE Ensor J Hooft L Altman DG . A guide to systematic review and meta-analysis of prognostic factor studies. BMJ. (2019) 364:k4597. 10.1136/bmj.k4597 30700442

[16] Page MJ McKenzie JE Bossuyt PM Boutron I Hoffmann TC Mulrow CD . The PRISMA 2020 statement: an updated guideline for reporting systematic reviews. BMJ. (2021) 372:n71. 33782057 10.1136/bmj.n71 PMC8005924

[17] Arabi A El Rassi R El-Hajj Fuleihan G. Hypovitaminosis D in developing countries-prevalence, risk factors and outcomes. Nat Rev Endocrinol. (2010) 6:550–61. 10.1038/nrendo.2010.146 20852586

[18] Graham EM Taylor SN Zyblewski SC Wolf B Bradley SM Hollis BW . Vitamin D status in neonates undergoing cardiac operations: relationship to cardiopulmonary bypass and association with outcomes. J Pediatr. (2013) 162:823–6. 10.1016/j.jpeds.2012.10.013 23149171 PMC3578094

[19] McNally JD Menon K Chakraborty P Fisher L Williams KA Al-Dirbashi OY . Impact of anesthesia and surgery for congenital heart disease on the vitamin d status of infants and children: a prospective longitudinal study. Anesthesiology. (2013) 119:71–80. 10.1097/ALN.0b013e31828ce817 23470437

[20] Sriram K Perumal K Alemzadeh G Osei A Voronov G. The relationship between immediate preoperative serum 25-hydroxy-vitamin D3 levels and cardiac function, dysglycemia, length of stay, and 30-d readmissions in cardiac surgery patients. Nutrition. (2015) 31:820–6. 10.1016/j.nut.2014.11.022 25721864

[21] Skuladottir GV Cohen A Arnar DO Hougaard DM Torfason B Palsson R Indridason OS. Plasma 25-hydroxyvitamin D2 and D3 levels and incidence of postoperative atrial fibrillation. J Nutr Sci. (2016) 5:e10. 10.1017/jns.2015.38 27066255 PMC4791515

[22] Shadvar K Ramezani F Sanaie S Maleki TE Arbat BK Nagipour B. Relationship between plasma level of vitamin D and post operative atrial fibrillation in patients undergoing CABG. Pak J Med Sci. (2016) 32:900–4. 10.12669/pjms.324.10587 27648036 PMC5017099

[23] Gode S Aksu T Demirel A Sunbul M Gul M Bakir I . Effect of vitamin D deficiency on the development of postoperative atrial fibrillation in coronary artery bypass patients. J Cardiovasc Thorac Res. (2016) 8:140–6. 10.15171/jcvtr.2016.29 28210468 PMC5304095

[24] Emren SV Aldemir M Ada F. Does deficiency of vitamin D increase new onset atrial fibrillation after coronary artery bypass grafting surgery? Heart Surg Forum. (2016) 19:E180–4. 10.1532/hsf.1531 27585197

[25] Abou Zahr R Faustino EVS Carpenter T Kirshbom P Hall EK Fahey JT . Vitamin D status after cardiopulmonary bypass in children with congenital heart disease. J Intensive Care Med. (2017) 32:508–13. 10.1177/0885066616652077 27251108

[26] Cerit L Kemal H Gulsen K Ozcem B Cerit Z Duygu H. Relationship between Vitamin D and the development of atrial fibrillation after on-pump coronary artery bypass graft surgery. Cardiovasc J Afr. (2017) 28:104–7. 10.5830/CVJA-2016-064 27701486 PMC5488049

[27] Özsin KK Sanri US Toktaş F Kahraman N Yavuz S. Effect of plasma level of vitamin D on postoperative atrial fibrillation in patients undergoing isolated coronary artery bypass grafting. Braz J Cardiovasc Surg. (2018) 33:217–23. 10.21470/1678-9741-2017-0214 30043913 PMC6089122

[28] Daie M Hajhossein Talasaz A Karimi A Gholami K Salehiomran A Ariannejad H . Relationship between vitamin D levels and the incidence of post coronary artery bypass graft surgery atrial fibrillation. J Tehran Heart Cent. (2018) 13:159–65. 10.18502/jthc.v13i4.639 30972113 PMC6450811

[29] Zajic P Heschl S Schörghuber M Srekl-Filzmaier P Stojakovic T Weixler V . Vitamin D assessment in perioperative medicine and critical care: a prospective observational pilot study. Wien Klin Wochenschr. (2019) 133:79–85. 10.1007/s00508-019-01584-x 31802221 PMC7875852

[30] Ney J Heyland DK Amrein K Marx G Grottke O Choudrakis M . The relevance of 25-hydroxyvitamin D and 1,25-dihydroxyvitamin D concentration for postoperative infections and postoperative organ dysfunctions in cardiac surgery patients: the eVIDenCe study. Clin Nutr. (2019) 38:2756–62. 10.1016/j.clnu.2018.11.033 30583965

[31] Dohain AM Almogati J Al-Radi OO Elassal AA Zaher ZF Fatani TH . Serum vitamin D status following pediatric cardiac surgery and association with clinical outcome. Eur J Pediatr. (2020) 179:635–43. 10.1007/s00431-019-03538-x 31865429

[32] Wells G Shea B O'Connell D Peterson J Welch Losos M Tugwell P . The Newcastle-Ottawa Scale (NOS) for Assessing the Quality of Nonrandomised Studies in Meta-Analyses (2014).

[33] Boivin J Griffiths E Venetis CA. Emotional distress in infertile women and failure of assisted reproductive technologies: meta-analysis of prospective psychosocial studies. BMJ. (2011) 342:d223. 10.1136/bmj.d223 21345903 PMC3043530

[34] Zarei M Najafi M Movahedi E Javanbakht MH Choi YH Yaseri M . The predictive role of circulating telomerase and vitamin D for long-term survival in patients undergoing coronary artery bypass grafting surgery (CABG). PLoS ONE. (2020) 15:e0237477. 10.1371/journal.pone.0237477 32790742 PMC7425905

[35] Obeid FA Yost G Bhat G Drever E Tatooles A. Effect of vitamin D level on clinical outcomes in patients undergoing left ventricular assist device implantation. Nutr Clin Pract. (2018) 33:825–30. 10.1002/ncp.10078 29603408

[36] Cerit L Özcem B Cerit Z Duygu H. Preventive effect of preoperative vitamin D supplementation on postoperative atrial fibrillation. Braz J Cardiovasc Surg. (2018) 33:347–52. 10.21470/1678-9741-2018-0014 30184031 PMC6122752

[37] Zittermann A Kuhn J Ernst JB Becker T Larisch J Dreier J . Circulating 25-Hydroxyvitamin D and 1,25-Dihydroxyvitamin D concentrations and postoperative infections in cardiac surgical patients: the CALCITOP-study. PLoS ONE. (2016) 11:e0158532. 10.1371/journal.pone.0158532 27355377 PMC4927161

[38] Dretzke J Ensor J Bayliss S Hodgkinson J Lordkipanidzé M Riley RD . Methodological issues and recommendations for systematic reviews of prognostic studies: an example from cardiovascular disease. Syst Rev. (2014) 3:140. 10.1186/2046-4053-3-140 25466903 PMC4265412

[39] Foroutan F Guyatt G Zuk V Vandvik PO Alba AC Mustafa R . GRADE guidelines 28: use of GRADE for the assessment of evidence about prognostic factors: rating certainty in identification of groups of patients with different absolute risks. J Clin Epidemiol. (2020) 121:62–70. 10.1016/j.jclinepi.2019.12.023 31982539

[40] Iorio A Spencer FA Falavigna M Alba C Lang E Burnand B . Use of GRADE for assessment of evidence about prognosis: rating confidence in estimates of event rates in broad categories of patients. BMJ. (2015) 350:h870. 10.1136/bmj.h870 25775931

[41] Wagner CL Greer FR American Academy of Pediatrics Section on B American Academy of Pediatrics Committee on N. Prevention of rickets and vitamin D deficiency in infants, children, and adolescents. Pediatrics. (2008) 122:1142–52. 10.1542/peds.2008-1862 18977996

[42] Brehm JM Celedon JC Soto-Quiros ME Avila L Hunninghake GM Forno E . Serum vitamin D levels and markers of severity of childhood asthma in Costa Rica. Am J Respir Crit Care Med. (2009) 179:765–71. 10.1164/rccm.200808-1361OC 19179486 PMC2675563

[43] Wayse V Yousafzai A Mogale K Filteau S. Association of subclinical vitamin D deficiency with severe acute lower respiratory infection in Indian children under 5 y. Eur J Clin Nutr. (2004) 58:563–7. 10.1038/sj.ejcn.1601845 15042122

[44] McNally JD Leis K Matheson LA Karuananyake C Sankaran K Rosenberg AM. Vitamin D deficiency in young children with severe acute lower respiratory infection. Pediatr Pulmonol. (2009) 44:981–8. 10.1002/ppul.21089 19746437

[45] Maiya S Sullivan I Allgrove J Yates R Malone M Brain C . Hypocalcaemia and vitamin D deficiency: an important, but preventable, cause of life-threatening infant heart failure. Heart. (2008) 94:581–4. 10.1136/hrt.2007.119792 17690157

[46] Madden K Feldman HA Smith EM Gordon CM Keisling SM Sullivan RM . Vitamin D deficiency in critically ill children. Pediatrics. (2012) 130:421–8. 10.1542/peds.2011-3328 22869836 PMC4074622

[47] Santillan GE Vazquez G Boland RL. Activation of a beta-adrenergic-sensitive signal transduction pathway by the secosteroid hormone 1,25-(OH)2-vitamin D3 in chick heart. J Mol Cell Cardiol. (1999) 31:1095–104. 10.1006/jmcc.1999.0942 10336847

[48] Green JJ Robinson DA Wilson GE Simpson RU Westfall MV. Calcitriol modulation of cardiac contractile performance via protein kinase C. J Mol Cell Cardiol. (2006) 41:350–9. 10.1016/j.yjmcc.2006.05.019 16815434

[49] Scragg R Sowers M Bell C. Serum 25-hydroxyvitamin D, ethnicity, and blood pressure in the third national health and nutrition examination survey. Am J Hypertens. (2007) 20:713–9. 10.1016/j.amjhyper.2007.01.017 17586404

[50] Snijder MB Lips P Seidell JC Visser M Deeg DJ Dekker JM . Vitamin D status and parathyroid hormone levels in relation to blood pressure: a population-based study in older men and women. J Intern Med. (2007) 261:558–65. 10.1111/j.1365-2796.2007.01778.x 17547711

[51] Thomas GN Hartaigh B Bosch JA Pilz S Loerbroks A Kleber ME . Vitamin D levels predict all-cause and cardiovascular disease mortality in subjects with the metabolic syndrome: the Ludwigshafen Risk and Cardiovascular Health (LURIC) Study. Diabetes Care. (2012) 35:1158–64. 10.2337/dc11-1714 22399697 PMC3329808

[52] Scragg R Sowers M Bell C Third National H Nutrition Examination S. Serum 25-hydroxyvitamin D, diabetes, and ethnicity in the Third National Health and Nutrition Examination Survey. Diabetes Care. (2004) 27:2813–8. 10.2337/diacare.27.12.2813 15562190

[53] Chen WR Qian YA Chen YD Shi Y Yin DW Wang H . The effects of low vitamin D on coronary artery disease. Heart Lung Circ. (2014) 23:314–9. 10.1016/j.hlc.2013.08.012 24161735

[54] Seker T Gur M Yuksel Kalkan G Kuloglu O Yildiz Koyunsever N Yildiray Sahin D . Serum 25-hydroxyvitamin D level and extent and complexity of coronary artery disease. J Clin Lab Anal. (2014) 28:52–8. 10.1002/jcla.21643 24375475 PMC6807537

[55] Öztürk S Öztürk I. Atrial fibrillation after cardiac surgery and preoperative vitamin D levels: a systematic review and meta-analysis. Turk Gogus Kalp Damar Cerrahisi Derg. (2020) 28:101–7. 10.5606/tgkdc.dergisi.2020.18387 32175149 PMC7067020

[56] Tasdighi E Hekmat M Beheshti M Baghaei R Mirhosseini SM Torbati P . Vitamin D treatment attenuates heart apoptosis after coronary artery bypass surgery: a double-blind, randomized, placebo-controlled clinical trial. J Cardiovasc Pharmacol Ther. (2020) 25:338–45. 10.1177/1074248420920495 32323557

