# Supplementary figures and images for: Association Between the Circulating Level of 25-Hydroxyvitamin D and Clinical Results After Cardiac Surgery: A Meta-Analysis and Systematic Review
Source: Front Cardiovasc Med. 2021 Nov 15;8:734504. doi: 10.3389/fcvm.2021.734504 (PMC8634434; doi:10.3389/fcvm.2021.734504)

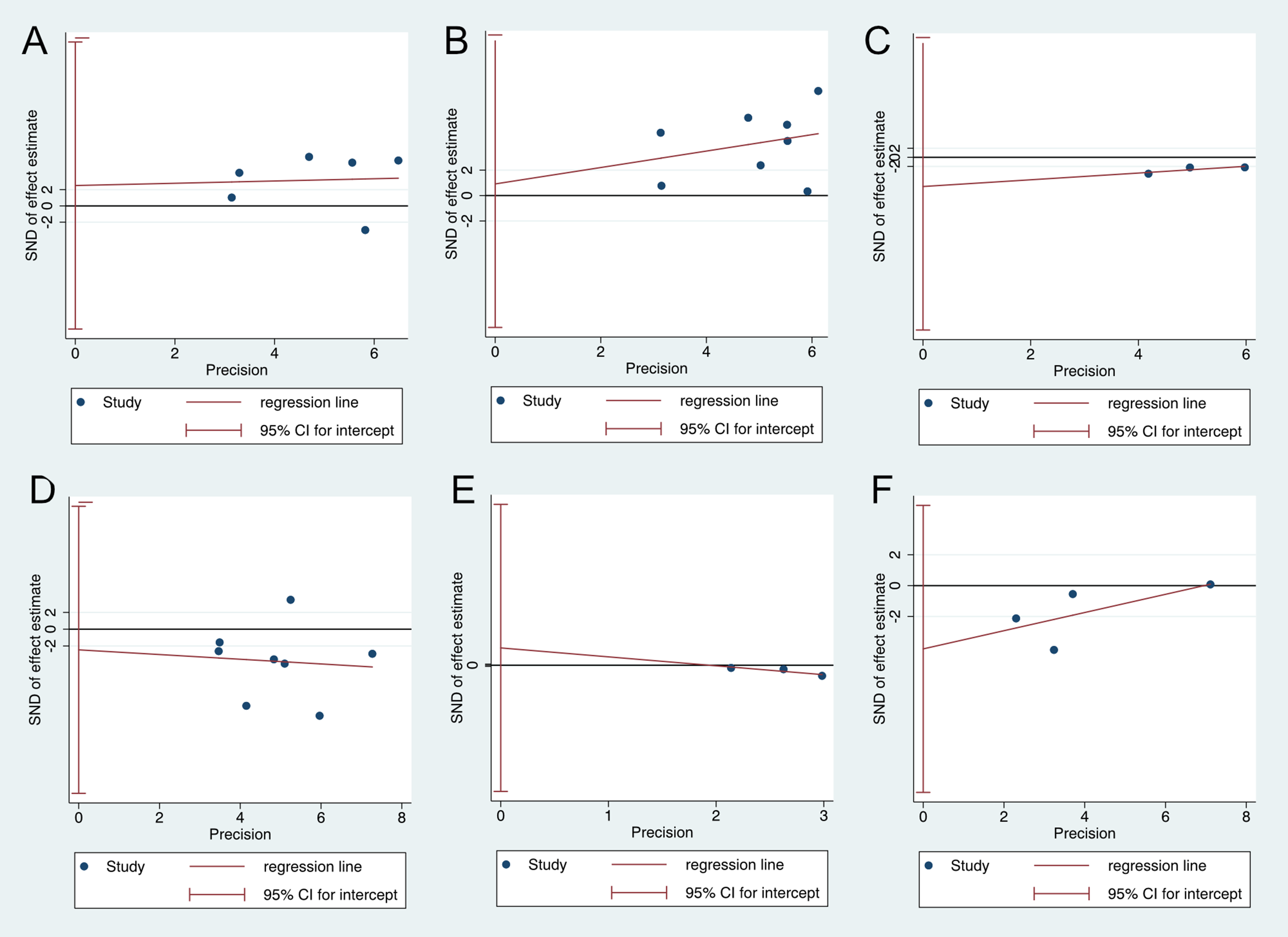

Supplement: Supplementary Figure 1 — Egger's publication bias plots for the assessment of potential publication bias. Each dot represents each study in the meta-analysis. (A) For the VitD level preoperative and immediate post cardiac surgery, and the Egger's plot did not show significant asymmetry (P = 0.713, t = 0.39, 95%CI (−15.13, 20.14), (B) for the VitD level preoperative and 24-h post cardiac surgery, the Egger's plot did not show marked asymmetry, P = 0.849, t = 0.2, 95%CI (−10.37, 12.21), (C) for the RRs of VitD deficiency rate of pre- and 24-h post-cardiac surgery, the Egger's plot did not show significant asymmetry, P =0.235, t = −2.58, 95%CI (−38.02, 25.19), (D) for the relationship between VitD level and severe events, the Egger's plot did not show significant asymmetry, P = 0.737, t = −0.35, 95%CI (−19.55, 14.64), (E) for the relationship between VitD level and maximum VIS, the Egger's plot did not show significant asymmetry, P = 0.368, t = 1.54, 95%CI (−221.27, 282.09), (F) for the relationship between VitD level and ICU stay duration. This Egger's plot indicates no publication bias with a P > 0.05. RR, risk ratio. CI, confidence interval. [file Image_1.TIFF]
